# Cephalomedullary Nail Lag Screw Migration Following Open Reduction and Internal Fixation of an Intertrochanteric Femur Fracture in a Patient With Body Mass Index of 63

**DOI:** 10.7759/cureus.69916

**Published:** 2024-09-22

**Authors:** Brody Scholl, Son Tran, Sumit Patel, Cameron J Vanlaningham, Joshua Veenstra

**Affiliations:** 1 Orthopaedic Surgery, Western Michigan University Homer Stryker M.D. School of Medicine, Kalamazoo, USA; 2 Trauma Surgery, Western Michigan University Homer Stryker M.D. School of Medicine, Kalamazoo, USA; 3 Sports Medicine, Western Michigan University Homer Stryker M.D. School of Medicine, Kalamazoo, USA

**Keywords:** cephalomedullary nail, hemi arthroplasty, intertrochanteric fracture, medial migration of lag screw, patients with a bmi ≥ 50, post-op complications

## Abstract

Hip fractures are prevalent among the elderly population and are correlated with notable morbidity and mortality. Treatment with intramedullary nails has risen in popularity with good results, however complications can occur. We report on a 62-year-old female with a body mass index of 63 kg/m^2^ who presents with cephalomedullary nail lag screw migration following an open reduction and internal fixation of an intertrochanteric fracture, which was subsequently converted to a hip hemiarthroplasty after hardware failure of her cephalomedullary nail. Proper radiographic follow-up is essential for catching the complication early and preventing further injury. Hemiarthroplasty is an appropriate next step when facing this complication.

## Introduction

Intertrochanteric femur fractures are one of the most common fracture patterns in the geriatric population [1]. Patients with this fracture are unable to bear weight on their extremity and are usually treated with open reduction and internal fixation within 24 hours of presenting [2]. These fractures are usually treated with a dynamic hip screw or intramedullary nailing as they are extracapsular and do not require arthroplasty [3]. In patients with intertrochanteric fractures, intramedullary nailing and compression through the fracture site with a lag screw is the gold standard treatment [4]. Complications from operative treatment with a cephalomedullary nail include hardware failure, nonunion, malunion, infection/hematoma, and avascular necrosis [5]. Patients with higher body mass index (BMI) tend to have an increased risk of complications, including respiratory symptoms, wound infection, and sepsis [6]. A multicenter study looking at the rate of complications following open reduction and internal fixation with cephalomedullary nails shows that medial migration of the lag screw accounts for 0.3% of all complications [7]. As such, it is a rare but known complication of this treatment. There have been previous case reports describing similar complications, however, this is the first time in the literature that the intrapelvic migration of cephalomedullary nail lag screw occurs in patients with a BMI >50.

## Case presentation

**Figure 1:**
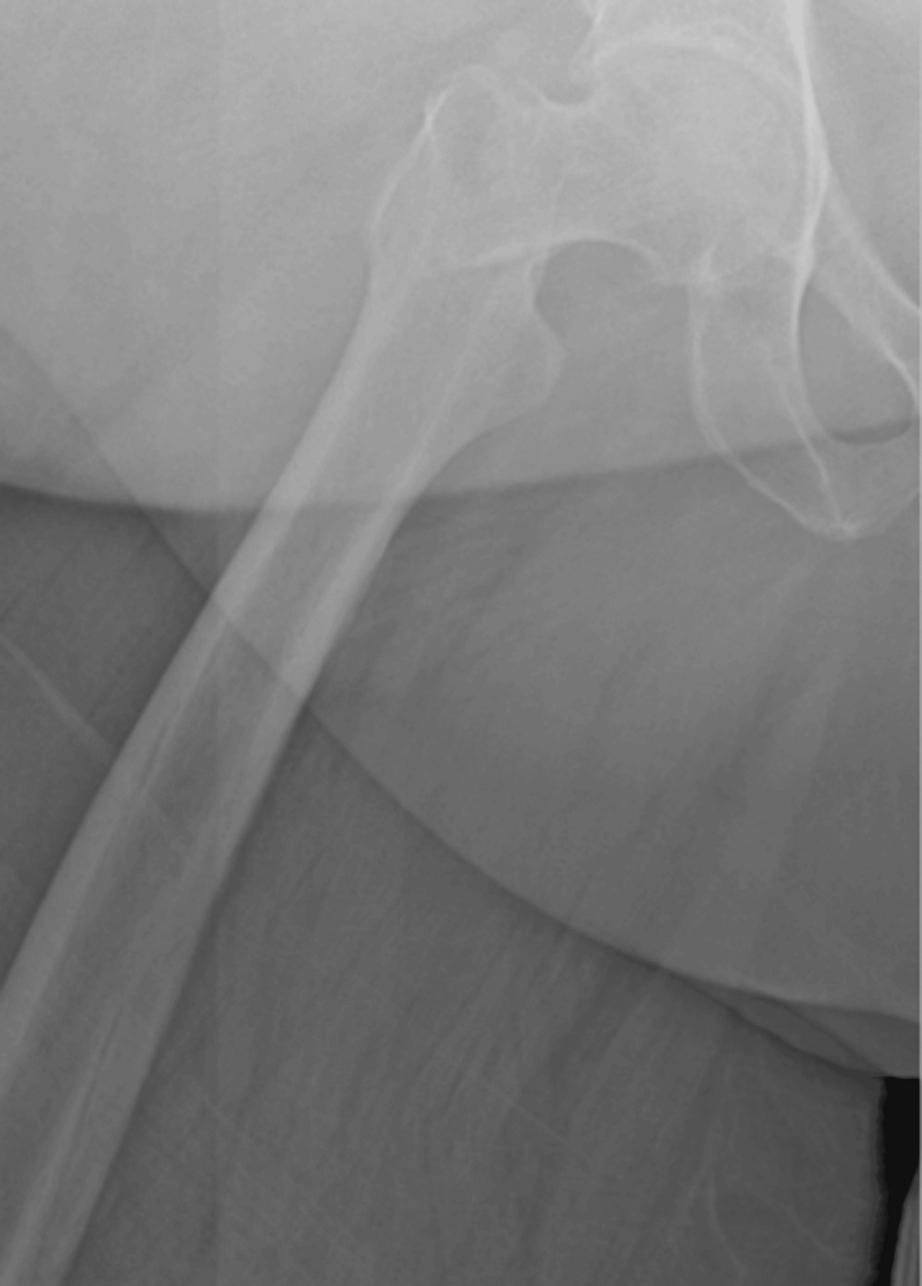
AP of right hip in emergency department after initial injury reveals a two-part displaced intertrochanteric femur fracture

**Figure 2:**
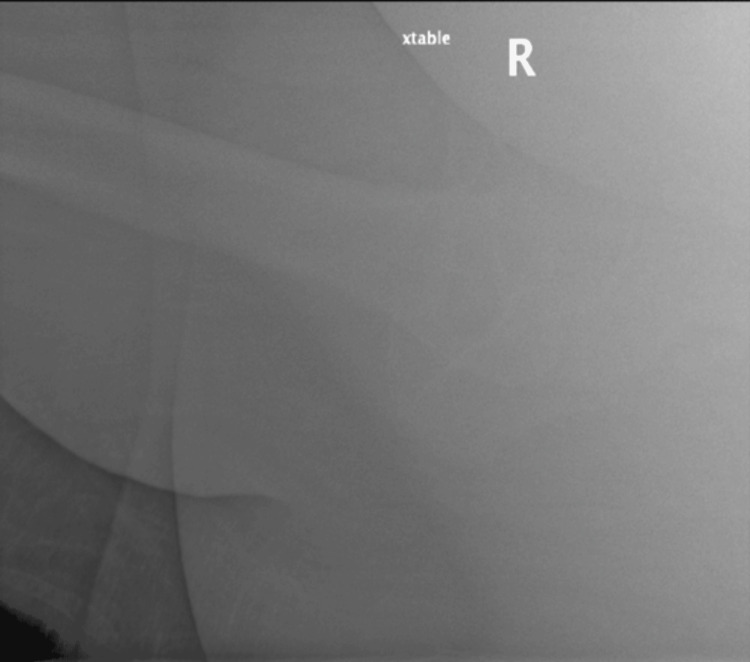
Attempted lateral of right hip in emergency department after initial injury reveals a two-part displaced intertrochanteric femur fracture

We report on the case of a 62-year-old female, who provided informed consent, with a past medical history of coronary artery disease, congestive heart failure, hyperlipidemia, obesity (BMI 63 kg/m2), and insulin-dependent type 2 diabetes mellitus was seen in the emergency department after a ground level fall. Additionally, the patient has a history of bilateral total knee arthroplasty completed 14 years prior on the left and six years prior on the right. Physical exam revealed a shortened and externally rotated right lower extremity with palpable distal pulses and intact sensation. Plain radiographs revealed a right two-part displaced intertrochanteric femur fracture (Figures 1, 2). Imaging was difficult to obtain in the emergency department due to the patient’s body habitus, however radiographs of the knee revealed proper placement of her bilateral knee prostheses. The patient was taken to the operating room the next day for open reduction and internal fixation of her fracture with a short cephalomedullary nail. Because of the patient’s weight (206 kg/454 lbs), she was unable to be placed on the traction bed for surgery as the limit is 400 pounds. She was placed on a surgical table in the lateral position with her right leg facing up. This made the procedure more difficult, as obtaining a reduction was more challenging and it was more difficult to obtain proper radiographs. A short nail was the implant of choice based on institution practice and the ease of implant placement secondary to the use of a fracture table and the limited options for patient positioning. The incisions made were much larger, as the patient’s size was greater than the standard aiming arm. Ultimately, appropriate reduction was obtained, and the nail and lag screw placement were confirmed with radiographs. The set screw was confirmed to have been placed with a tip-apex distance of 7.9 millimeters and all wounds were irrigated and closed in standard layered fashion. The patient was given three doses of cefazolin 3 grams every eight hours and a deep vein prophylaxis regimen of enoxaparin while inpatient and acetylsalicylic acid 325 milligrams once daily for 30 days after discharge. The patient was made non-weight bearing on her right lower extremity due to her weight for the first two weeks. She had difficulties mobilizing with physical therapy after the surgery. Physical therapy recommended that she be discharged to a low-intensity rehabilitation, and she was discharged on postoperative day two. 

**Figure 3:**
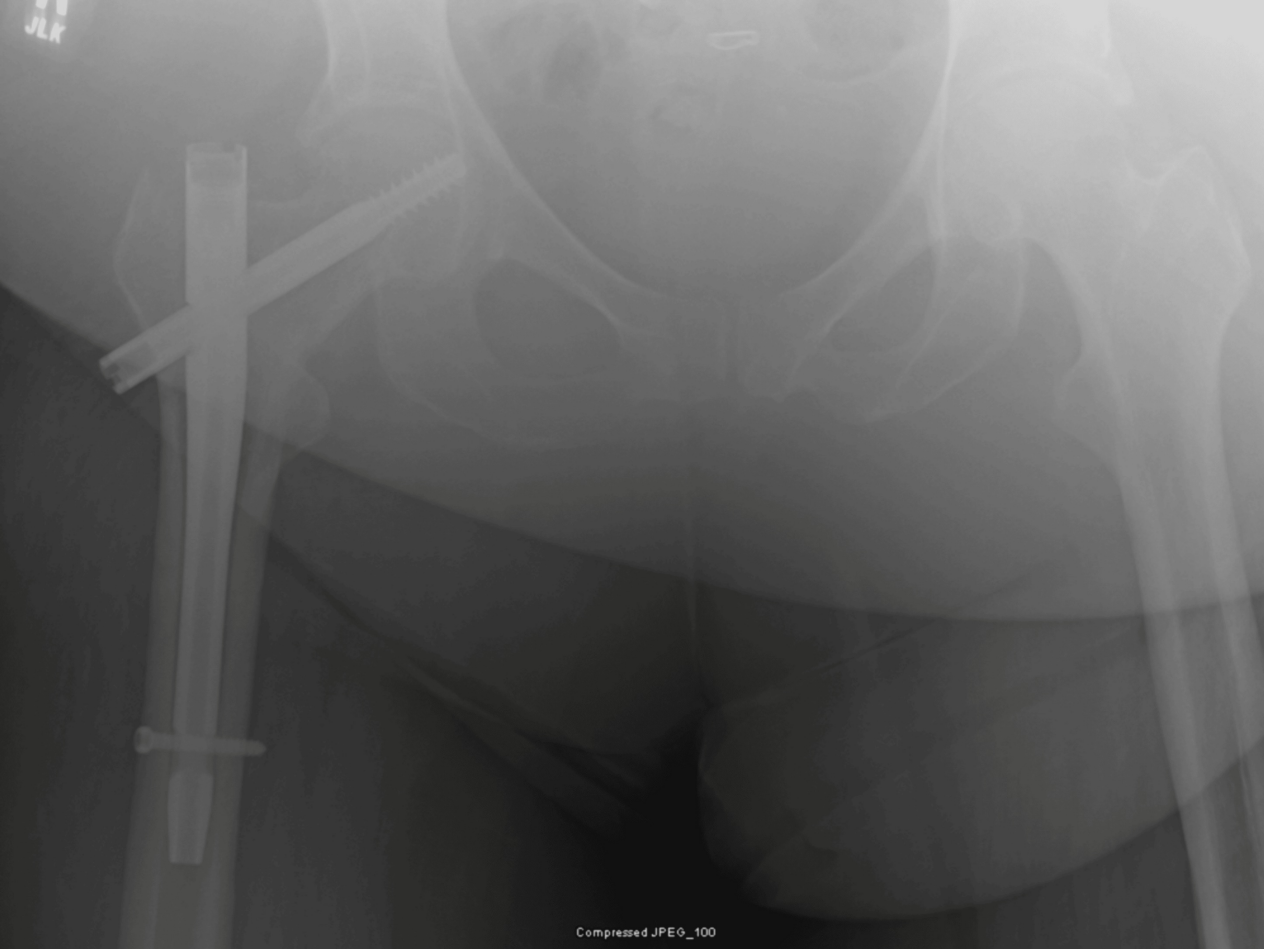
Films obtained two weeks postoperatively show AP of the pelvis with proper placement of implants and a calculated tip-apex distance of 7.9 mm

**Figure 4:**
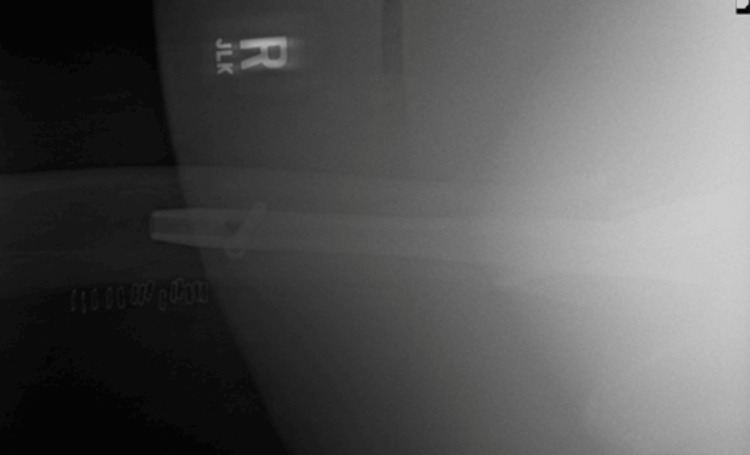
Lateral pelvis of right hip of patient two weeks postoperatively with proper implant positioning

The patient's first clinic visit two weeks after the index procedure showed a well-healed incision and radiograph showed proper placement of the implants with a calculated tip-apex distance of 7.9 millimeters (Figures 3, 4). She was made toe-touch weight bearing and instructed to follow up again at six weeks postoperatively. The patient did not present for her six-week postoperative follow-up at which time the plan would have been to begin weight bearing as tolerated. Rehabilitation was complicated by a urinary tract infection that was treated with trimethoprim/sulfamethoxazole. Patient’s strength continued to improve, and she was able to stand and pivot to a wheelchair. Six weeks after surgery, the patient was discharged from rehabilitation with home health physical therapy. 

**Figure 5:**
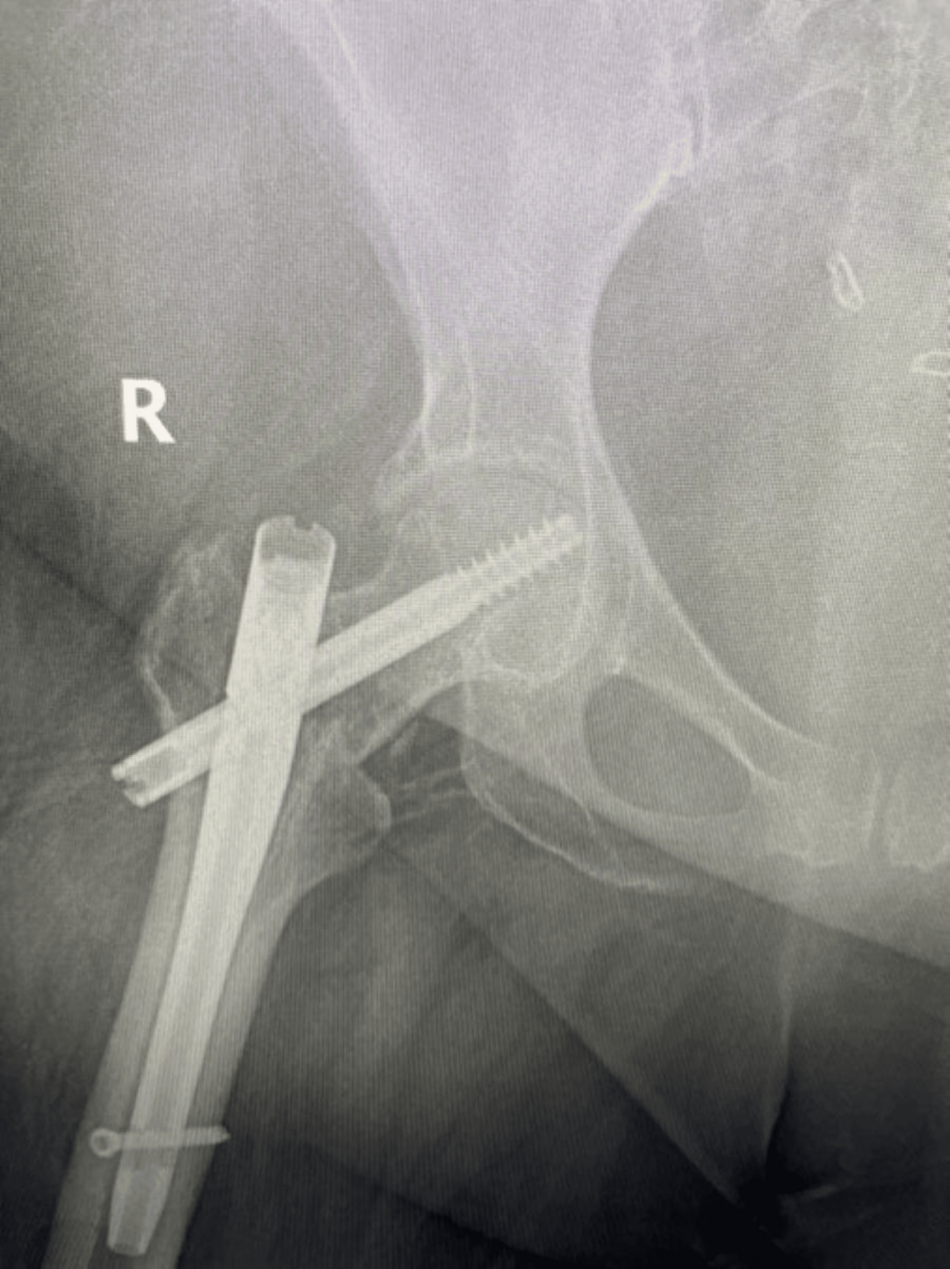
AP of right hip of patient at two months postoperatively and after a ground-level fall shows no bony pathology and appropriate hardware positioning

Eight weeks after surgery, the patient presented to the emergency department after a ground-level fall. She stated that her walker had slid out from underneath her and reported right hip and knee pain. Plain radiographs were obtained and showed no acute bony pathology with appropriate positioning of her hardware (Figure 5). Given that the patient was able to ambulate adequately, further imaging was not ordered and the patient was discharged with a one-week supply of Norco and instructed to follow up with her primary care physician and orthopaedics, but the patient failed to follow up with her orthopaedic surgeon. 

**Figure 6:**
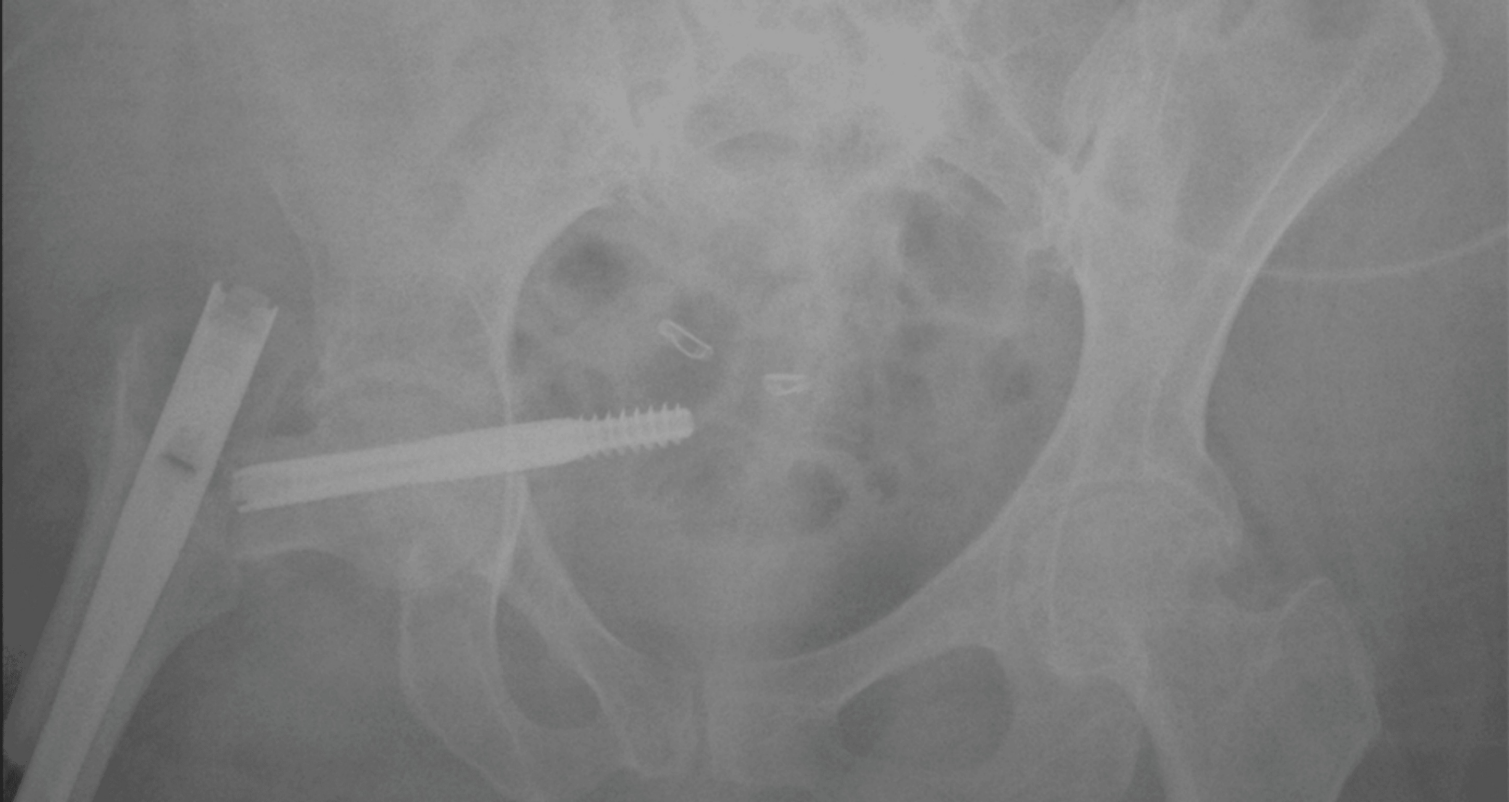
AP Pelvis demonstrating intrapelvic migration of lag screw at four months postoperatively

**Figure 7:**
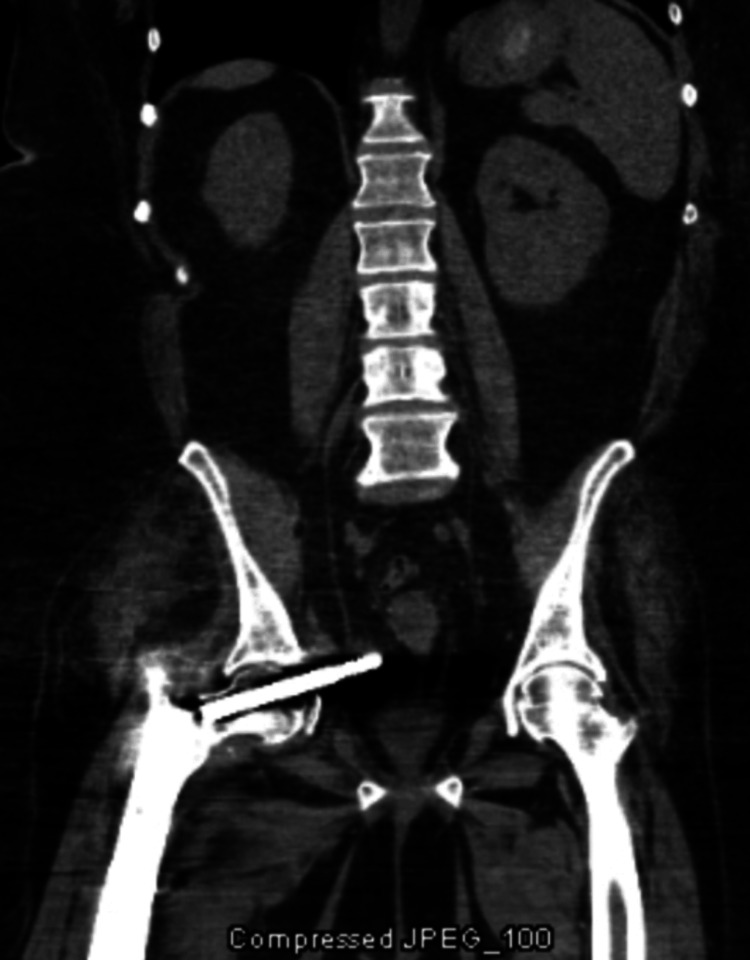
CT coronal view demonstrating intrapelvic migration of the lag screw abutting against the rectum and vagina

**Figure 8:**
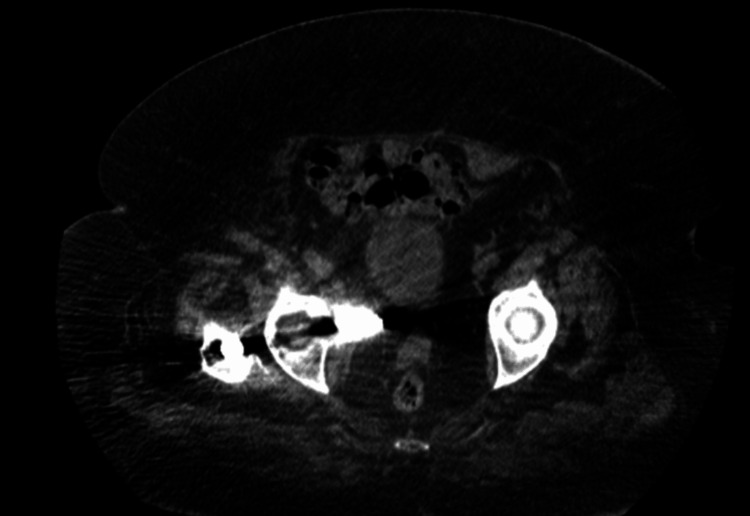
CT axial view demonstrating intrapelvic migration of the lag screw abutting against the rectum and vagina

Three months after the index procedure, the patient presented to her family health center for a follow-up appointment. At this time, the patient endorsed one week of right hip pain with a burning sensation, and she was unable to bear weight. Patient denied any inciting traumatic event. She was transferred to the emergency department for further evaluation which noted that the hardware was in proper position, but she had another urinary tract infection and was discharged home with Bactrim. Four months after cephalomedullary nail placement, the patient presented to the emergency department with increased pain and inability to bear weight. Plain radiographs demonstrated intrapelvic migration of the lag screw of the gamma nail through the acetabulum and a non-union at the fracture site (Figure 6). A CT scan was obtained which confirmed the findings and confirmed that there was no intrapelvic injury or hematoma, but there was mass effect on the rectum and vagina from the lag screw (Figures 7, 8). At this point, it was determined that the hardware would be removed, and her right hip would be converted to a hemiarthroplasty. A hemiarthroplasty was chosen for revision based on the patient's comorbidities (morbid obesity, diabetes, hypertension, congestive heart failure, and coronary artery disease status post placement of five stents), mobility status as requiring an assistive device, and intraoperative determination of an unstable acetabulum given the defect from the lag screw.

**Figure 9:**
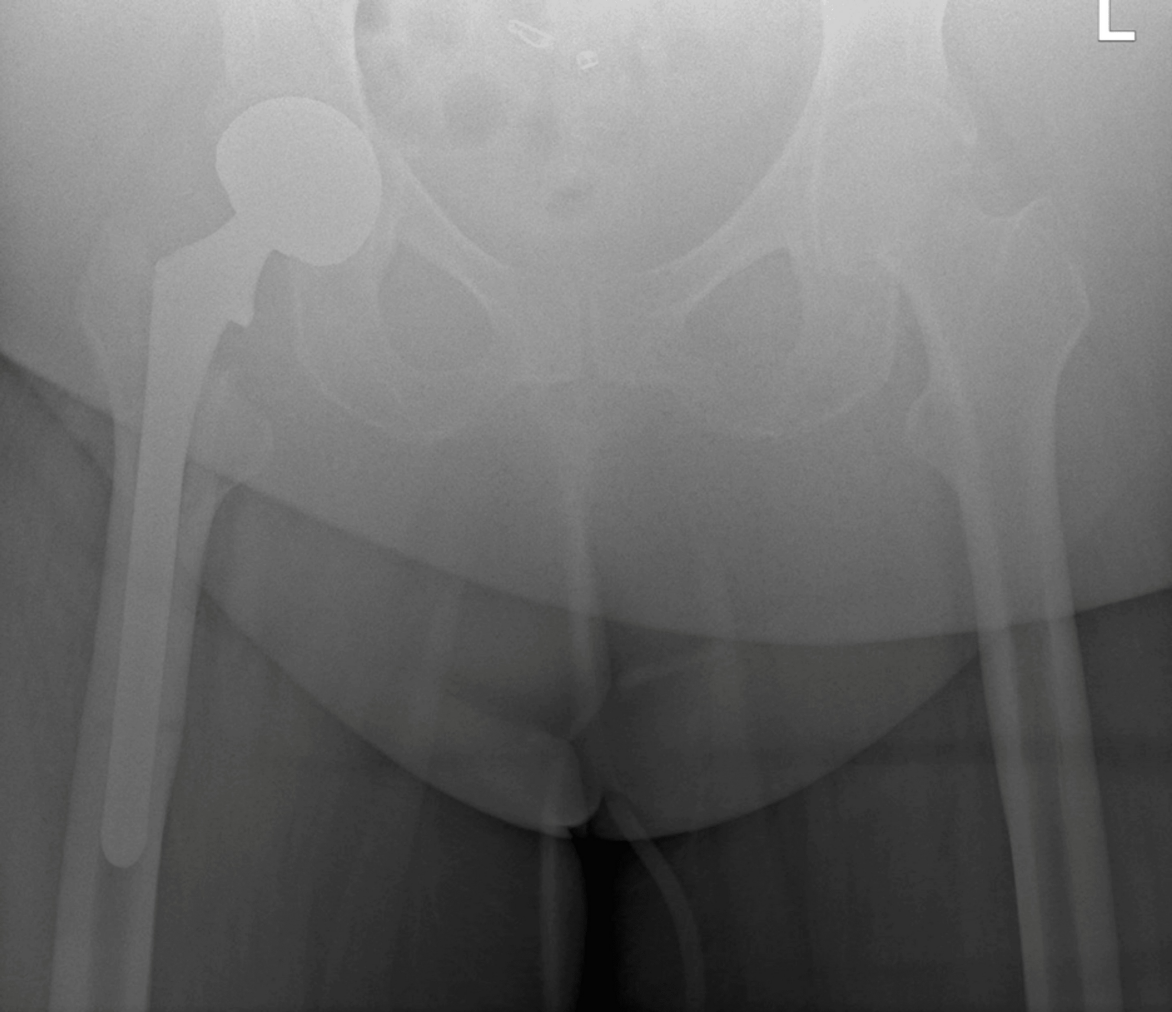
AP of right hip immediately postoperatively following right hip hemiarthroplasty reveals proper implant placement

**Figure 10:**
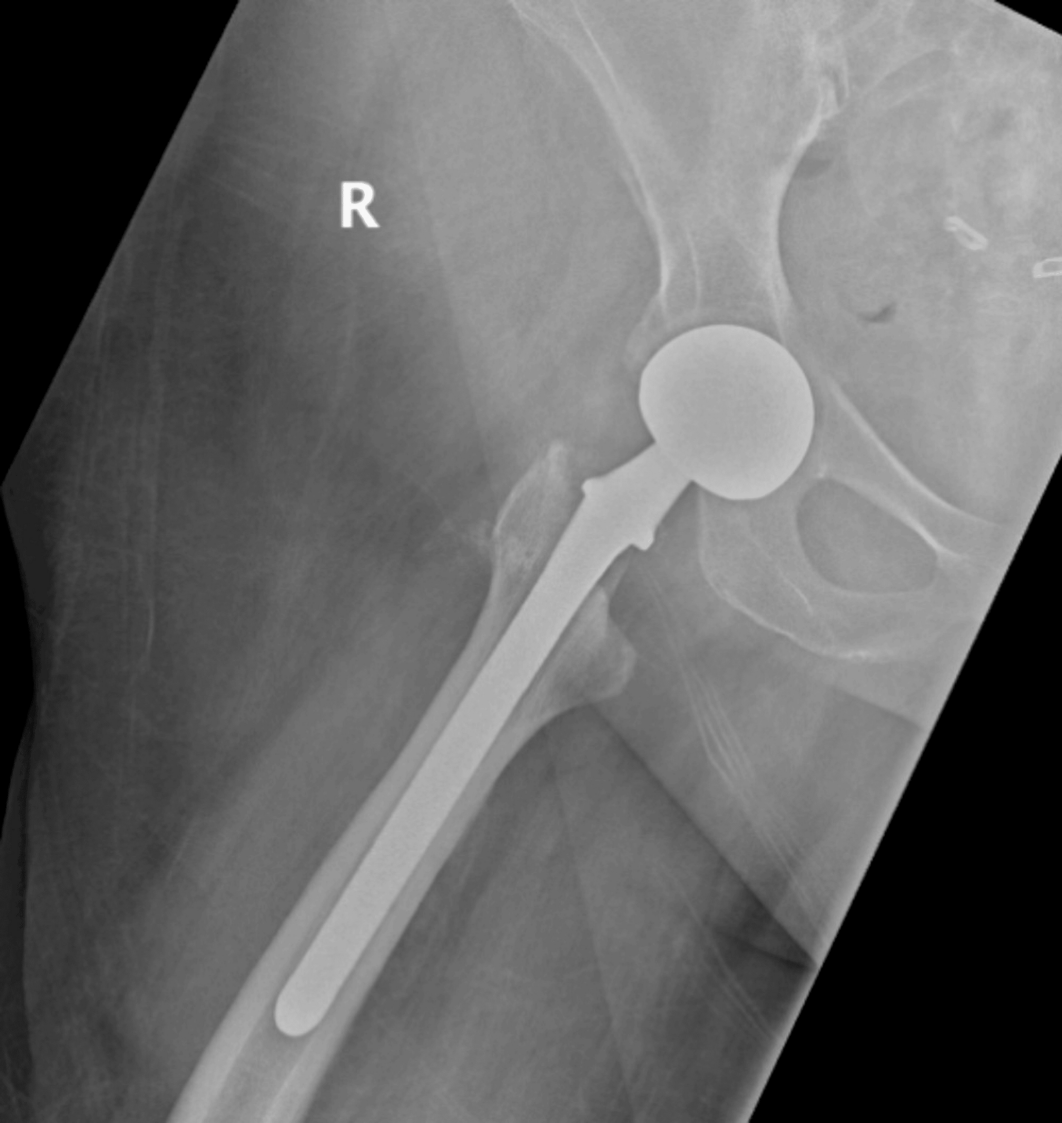
Lateral of right hip at two months postoperatively following right hip hemiarthroplasty reveals proper implant placement

Intraoperatively, there was no adherence of pelvic structures to the lag screw as it was being removed. A defect the size of a nickel was palpated in the acetabulum and there were no apparent signs visually or by palpation of defect extension anterior, posterior, superior, or inferior. The superior dome, posterior wall and column, and the anterior wall and column were palpable and intact. A hemiarthroplasty was performed at this time and the procedure had no complications. A stem was placed that bypassed the interlocking screw hole by 2 inches. Radiographs immediately postoperatively showed proper implant placement (Figure 9). The patient was made weight-bearing as tolerated up to 10 feet for transfers postoperatively instead of weight-bearing as tolerated due to her body habitus. The patient was able to stand with physical therapy the day following surgery and was discharged to inpatient rehabilitation in stable condition after seven days. Clinical follow-up two months after the index procedure showed a well-healed incision and radiographs showed proper placement of the implants (Figure 10).

## Discussion

Medial migration of the lag screw after internal fixation with a Gamma 3 nail has been described in previous case reports (Table 1) [8-17]. However, this is the first case described of lag screw migration in a morbidly obese patient who had proper fracture reduction postoperatively and confirmation of set screw placement. A previous case report described by Werner-Tutshcku et al. has cited the “Z” effect as a potential mechanism of instrumentation failure [18]. The “Z” effect describes medial sliding of the lag screw due to varus loading stress during weight bearing. Certain fracture patterns can also lead to toggling of the screw inside of the femoral head. This patient has a morbidly obese body habitus with a BMI of 63 kg/m2. This means that weight-bearing stress is significantly higher in her than in other patients and will likely produce a varus load which results in excess toggling of the lag screw that increases the chances of hardware failure. Given the adequate reduction and stable positioning of the implant up to three months postoperatively, it is unlikely that failure was due to inadequate reduction or surgeon error with failure of placing the setscrew. Therefore, hardware failure in this case is most likely due to the patient’s obese body habitus.

**Table 1:** Case studies of medial migration of the lag screw after internal fixation of an intertrochanteric femur fracture

Author	Sex/Age	Fracture	Days before medial screw migration was seen/extent of migration	Revision surgery	Outcome	Author perspective on causes
Thein et al. (2014) [8]	Female/69	A class IV reverse intertrochanteric femur fracture	5/touches a branch of the internal iliac artery	Total hip arthroplasty	Discharge home after 4 weeks, 6-month follow-up radiograph shows uneventful healing	An imperfect reduction and lag screw placed too high and anterior to the femoral head; however, the tip-apex-diameter (TAD) was <24 mm. The set screw was not placed correctly in one of the four grooves of the lag screw shaft, which could lead to its migration
Li et al. (2010) [9]	Female/77	Intertrochanteric fracture with lesser trochanter separation	70/through the medial wall of the acetabulum, patient notes no pain and is able to ambulate	Lag screw was removed, and a shorter one was placed	Returned to full weight bearing within 1 year and ambulation with a cane	Poor device placement and unstable fracture patterns
Takasago et al. (2014) [17]	Female/63	Trochanteric fracture in the left femur, AO type 31-A1.2	42/deep in the pelvis, between the internal and external iliac vessels, and tangent to the bladder and the sigmoid	Lag screw was removed, 3 weeks later, total hip arthroplasty	Uneventful. 2-year follow-up shows return to normal activity	The set screw not being inserted appropriately to control the lag screw; the lag screw was not locked rotationally and thus could not prevent spinning of the femoral head and insufficient reduction.
Heinman et al. (2010) [10]	Female/83	Unstable intertrochanteric	21/intra-abdominal space	Lag screw removed, 2 weeks later total hip arthroplasty	1 day after surgery, hip dislocated, treated with reduction and mobilizing cast for 6 weeks, then hip brace for 6 weeks. Able to mobilize independently after 4 months	“Z” effect, unstable fractures, not locking the set screw enough
Van Hoef (2016) [11]	Female/81	Peritrochanteric multifragmentary fracture of the proximal femur, AO Classification 31-A2	42/into the acetabulum	Lag screw was removed, replaced with a cemented hip arthroplasty	Discharge after 15 days to rehab	The set screw was not inserted correctly or it might have loosened over time
Flint et al. (2010) [12]	Female/82	Unstable intertrochanteric fracture with reverse obliquity subtrochanteric extension	112/near the external iliac artery and vein	Lag screw removed, replaced with an uncemented hip arthroplasty	Weight-bearing immediately and discharge to rehab after 7 days	Over-reaming the femoral head, persistent fracture instability
Lucke et al. (2009) [13]	Male/75	Unstable peritrochanteric femur fracture (AO A2)	3/5 cm from the acetabulum and into the pelvis	Removal of the nail and lag screw followed by placement of a cementless, bipolar, long-stem prosthesis	One year postoperatively, the patient is fully mobile	Toggling of the nail in an unstable fracture
Lucke et al. (2009) [13]	Male/68	Multifragmentary traumatic intertrochanteric femur fracture (AO A3.3)	180/adjacent to the posterior bladder wall	Lag screw removal, and a cemented bipolar prosthesis	Discharge after 14 days, but died 6 months after from other co-morbidities	Toggling of the nail in an unstable fracture
Tauber and Resch (2005) [14]	Female/84	Unstable trochanteric fracture (type A2.3)	56/tangent to the bladder	Lag screw removed, cemented hip arthroplasty	Complicated by infection, perforation of the sigmoid, necrotic muscle abscess and fistulas	No explanations
Pawar et al. (2020) [15]	Female/77	Comminuted intertrochanteric fracture	240/adjacent to the internal iliac	Embolized the internal iliac during the lag screw removal and hip arthroplasty to prevent bleeding	Discharge home on post-op day 3	Varus malreduction since patient had a previous contralateral fixation of the femoral neck, leading to the screw to cut out the femoral head and the set screw was not set appropriately
Lozano-Alvarez et al. (2013) [19]	Female/87	Subtrochanteric extension was seen (OTA 31-A2.3)	120/into varus	Lag screw removal, no hip arthroplasty due to patient co-morbidities	1 year after surgery, patient had complete varus collapse and ambulating on 2 crutches	“Z” effect
Lozano-Alvarez et al. (2013) [19]	Female/75	4-part intertro-chanteric fracture of the right femur (OTA 31-A2.3)	210/intrapelvic	Lag screw removal and total hip arthroplasty	No pain, ambulating with walker	“Z” effect
Fredj et al. (2022) [16]	Female/90	Intertrochanteric fracture (AO/OTA 31-A2)	2 years/through the acetabulum	Lag screw removed and cemented hemiarthroplasty was performed	-	-

It has been noted in the literature that medial migration of the lag screw can occur as soon as five days and up to three years after surgery [8-17]. Earlier onset of failure suggests imperfect reduction or that the set screw was not placed appropriately, leading to a rapid migration of the lag screw. Patients with later presentations of lag screw migration were more likely due to the “Z” effect phenomenon. Most cases in the literature suggest hardware removal and conversion to a total hip arthroplasty or hemiarthroplasty. One case described lag screw removal without arthroplasty and the patient was able to ambulate with crutches [19].

## Conclusions

To ensure an optimal outcome, proper reduction and ensuring placement of the set screw decreases the chance of medial migration of the lag screw. In patients with higher BMI and/or patients with complex fracture patterns, careful observation and slow transition to weight bearing is imperative, as well as close follow-up with radiographs to ensure proper placement of the implants, and to confirm fracture union. If the hardware were to fail, conversion to hemiarthroplasty or total hip arthroplasty have both been shown to have good patient outcomes.
